# NLRP3 inflammasome and NLRP3-related autoinflammatory diseases: From cryopyrin function to targeted therapies

**DOI:** 10.3389/fimmu.2022.1007705

**Published:** 2022-10-06

**Authors:** Chiara Moltrasio, Maurizio Romagnuolo, Angelo Valerio Marzano

**Affiliations:** ^1^ Dermatology Unit, Fondazione IRCCS Ca’ Granda Ospedale Maggiore Policlinico, Milan, Italy; ^2^ Department of Medical Surgical and Health Sciences, University of Trieste, Trieste, Italy; ^3^ Department of Pathophysiology and Transplantation, Università degli Studi di Milano, Milan, Italy

**Keywords:** inflammasome, NLRP3, cryopyrin associated periodic syndrome, gain-of-functions, mutations, anti-IL-1β treatments, deafness autosomal dominant 34, keratitis fugax hereditaria

## Abstract

The NLRP3 inflammasome is one of the NOD-like receptor family members with the most functional characterization and acts as a key player in innate immune system, participating in several physiological processes including, among others, the modulation of the immune system response and the coordination of host defences. Activation of the inflammasome is a crucial signaling mechanism that promotes both an acute and a chronic inflammatory response, which can accelerate the production of pro-inflammatory cytokines, mainly Interleukin (IL)-1β and IL-18, leading to an exacerbated inflammatory network. Cryopyrin associated periodic syndrome (CAPS) is a rare inherited autoinflammatory disorder, clinically characterized by cutaneous and systemic, musculoskeletal, and central nervous system inflammation. Gain-of-function mutations in *NLRP3* gene are causative of signs and inflammatory symptoms in CAPS patients, in which an abnormal activation of the NLRP3 inflammasome, resulting in an inappropriate release of IL-1β and gasdermin-D-dependent pyroptosis, has been demonstrated both in *in vitro* and in *ex vivo* studies. During recent years, two new hereditary NLRP3-related disorders have been described, deafness autosomal dominant 34 (DFN34) and keratitis fugax hereditaria (KFH), with an exclusive cochlear- and anterior eye- restricted autoinflammation, respectively, and caused by mutations in *NLRP3* gene, thus expanding the clinical and genetic spectrum of NLRP3-associated autoinflammatory diseases. Several crucial mechanisms involved in the control of activation and regulation of the NLRP3 inflammasome have been identified and researchers took advantage of this to develop novel target therapies with a significant improvement of clinical signs and symptoms of NLRP3-associated diseases. This review provides a broad overview of NLRP3 inflammasome biology with particular emphasis on CAPS, whose clinical, genetic, and therapeutic aspects will be explored in depth. The latest evidence on two “new” diseases, DFN34 and KFH, caused by mutations in *NLRP3* is also described.

## Introduction

The term inflammasome was introduced by Martinon et al., in 2002 to describe an intracellular high-molecular-weight complex that mediates the activation of inflammatory caspases (1). Inflammasomes are multimeric protein complexes, known as first responders in the innate immune response, sensing a wide spectrum of pathogens (pathogen-associated molecular patterns), stress, and damage signals (damage-associated molecular patterns), and subsequently promoting an inflammatory cell signaling with the production and release of pro-inflammatory cytokines and a form of programmed cell death (PCD), called pyroptosis (2). Inflammasomes are molecular regulators, whose responsiveness to stimuli is represented by their sensor molecule, which comprise a member of the pattern recognition receptors (PRR) system (3). To date, the five most studied inflammasomes are: i) nucleotide-binding and oligomerization (NOD) like receptor (NLR) containing pyrin domain (NLRP) 1; ii) NLRP3, iii) NLR caspase activation recruitment (CARD) domain-containing 4 (NLRC4), iv) absent in melanoma 2 (AIM2), and v) Pyrin inflammasomes (4). However, other members of PRRs with site-specific expression, including NLRP2 (5), NLRP6 (6), NLRP7 (7), NLRP12 (8), and IFI16 (9) have been also reported to act as sensors in inflammasome platform, playing an important role in the modulation of the innate immune response. Inflammasomes participate in several physiological processes including the orchestration of the immune system response, the coordination of host defences and the mediation of insulin signaling (10). Inflammasomes and their components can be also involved in panoptosis, defined as a unique inflammatory cell death pathway that integrates components from other cell death pathways, like pyroptosis, apoptosis and/or necroptosis and is implicated in driving innate immune responses and inflammation as well as homeostasis control (11). Being so important for tissue homeostasis, inflammasomes dysfunction has been associated to a variety of inflammatory, autoimmune, and infectious states, as well as degenerative and metabolic diseases and tumorigenesis, with specific inflammasomes involved in different diseases and several inflammasomes being activated even in the same disorder (2). These alterations can be also the fruit of genetic changes, as observed in cryopyrin associated periodic syndrome (CAPS), in which gain-of-functions mutations in *NLRP3* gene, encoding NLRP3 inflammasome, lead to its spontaneous formation in the absence of the activating signals, thus resulting in an exacerbated release of pro-inflammatory cytokines and CAPS related inflammatory symptoms (12).

CAPS defines a group of autoinflammatory diseases, comprising three conditions on a continuum of severity: familial cold auto-inflammatory syndrome (FCAS), Muckle–Wells syndrome (MWS) and chronic infantile neurological cutaneous articular (CINCA) syndrome, that is also called neonatal-onset multisystem inflammatory disorder (NOMID) (13). During recent years, two new hereditary NLRP3-related disorders have been described, deafness autosomal dominant 34 (DFN34) and keratitis fugax hereditaria (KFH), with an exclusive cochlear- and anterior eye- restricted autoinflammation, respectively, and caused by mutations in *NLRP3* gene (14, 15).

Given these associations, inflammasome inhibition has been regarded as a useful therapeutic target to improve symptoms and signs of these diseases.

As the role of the NLRP3 inflammasome pathway has been thoroughly studied in autoinflammatory conditions, in this review we will touch upon relevant features of the NLRP3 inflammasome activation process, discuss the clinical features of CAPS and the role of NLRP3 dysfunction in this latter, review the efficacy of therapies for CAPS, describe potential novel therapeutic targets whereby clinical trials are ongoing and briefly discuss some of the newly emerging molecules targeting NLRP3 inflammasome which have been assessed in CAPS mouse models. Additionally, we will give a brief overview of other autoinflammatory diseases in which *NLRP3* mutations are causative to the clinical phenotype (**Tables 1**, **2**).

**Table 1:** Main clinical symptoms and organ involvement of NLRP3-associated autoinflammatory diseases.

	FCAS	MWS	CINCA/NOMID	DFN34	KFH
**Attack pattern**	Recurrent Lasting <24 hours Precipitated by cold exposure	Recurrent Lasting 1 to 3 days	Recurrent-to-continuous 1-to-3 flares per day	Sub-clinical Progressive	Recurrent, unilateral Lasting 24-48 hours Improvement with age
**Urticaria-like rash** **(during the attacks)**	+	+	+	–	–
**Fever/chills/arthralgias** **(during the attacks)**	+	+	+	–	–
**Headache** **(during the attacks)**	+	+	+	–	–
**Amyloidosis**	Rare	+ (late-onset)	–	–	–
**Sensorineural hearing loss**	Rare	+ (progressive)	Yes	+ (bilateral, progressive)	–
**Ocular symptoms**	Keratitis- conjunctivitis	Keratitis- conjunctivitis	Anterior Uveitis Visual loss (due to chronic papilledema)	–	Keratitis Photophobia Peri-corneal and peri-conjunctival injection Red and painful eye Blurry vision after the attacks Reduced visual acuity (long-term)
**CNS involvement**	–	–	Chronic aseptic meningitis Hydrocephalus Papilledema Intellectual disability	–	–
**Osteoarticular involvement**	–	–	Destructive arthropathy	–	–

FCAS, Familial cold autoinflammatory syndrome; MWS Muckle Wells Syndrome; CINCA/NOMID, chronic infantile neurologic, cutaneous, and articular syndrome/neonatal-onset multisystem inflammatory disease; DFN34, Deafness Autosomal Dominant 34; KFH, Keratitis Fugax Hereditaria; CNS, Central nervous system. The symbol “+” corresponds to the presence of clinical features, while the symbol “-” corresponds to their absence.

**Table 2:** Brief overview of NLRP3-associated autoinflammatory diseases.

	FCAS	MWS	CINCA/NOMID	DFN34	KFH
**Estimated Prevalence**	1-2 per million inhabitants in USA 1 per 360.000 in Europe (France)	1-2 per million inhabitants in USA 1 per 360.000 in Europe (France)	1-2 per million inhabitants in USA 1 per 360.000 in Europe (France)	Only reported in two pedigrees worldwide	Only reported in seven Finnish families (Founder effect)
**Age at onset**	< 6 months	< 6 months	Congenital	Middle age	Second decade of life
**Inheritance pattern**	AD	AD *De novo*	AD *De novo*	AD	AD
**Prognosis**	Good	Moderate (depending on renal amyloidosis)	Severe	Good	Good

FCAS, Familial cold autoinflammatory syndrome; MWS Muckle Wells Syndrome**;** CINCA/NOMID, chronic infantile neurologic, cutaneous, and articular syndrome/neonatal-onset multisystem inflammatory disease; DFN34, Deafness Autosomal Dominant 34; KFH, Keratitis Fugax Hereditaria; AD, autosomal dominant.

## NLRP3 inflammasome biology

### Structure and function

**Figure 1:**
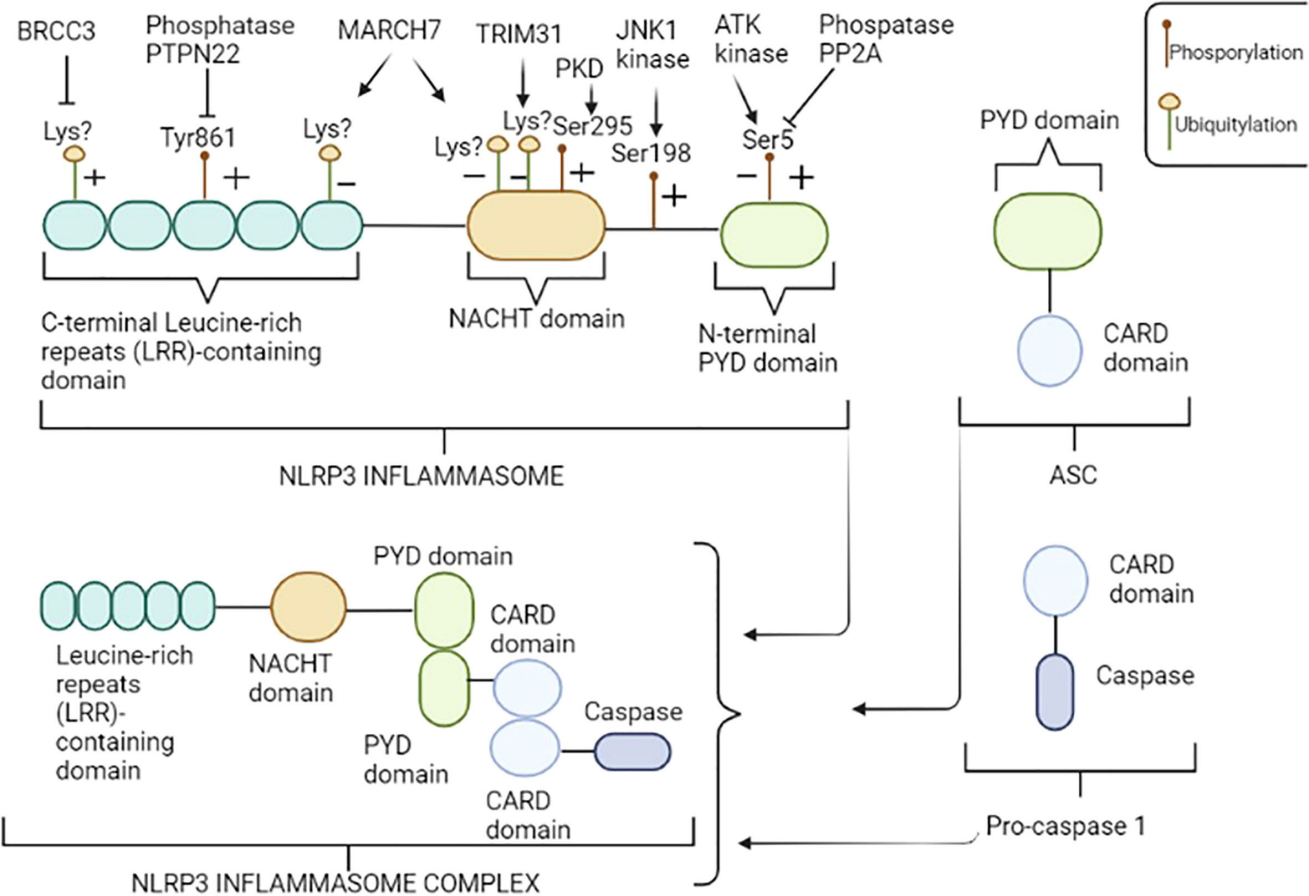
NLRP3 inflammasome structure. NLRP3 consists of three regions: the pyrin domain (PYD) in the N-terminal tail, the NACHT central domain and the leucine-rich repeats (LRR) containing domain in the C-terminal tail. NLRP3 inflammasome is a multimeric complex composed by NLRP3, ASC, and pro-caspase 1. NLRP3 recruits ASC through PYD-PYD interactions. In turn, pro-caspase 1 is recruited by ASC through CARD-CARD interactions to form the NLRP3-ASC-pro-caspase 1 inflammasome complex. NLRP3 is regulated at the posttranslational level by several modifications including, among others, phosphorylation and ubiquitylation. An illustrative image has been provided; enzyme “+” indicates that the enzyme promotes the activation of NLRP3 inflammasome, dephosphorylating and/or deubiquitylating specific residues, while “−” indicates repression of NLRP3 function, promoting phosphorylation and/or ubiquitylation of specific residues. Amino acid residues shown above refer to human NLRP3. Figure has been created with BioRender.com.

Among inflammasomes, NLRP3 – also known as cryopyrin - is the most widely and comprehensively characterized. It is a multimeric complex composed of a variety of proteins, in particular, comprising of the receptor protein NLRP3, that acts as a sensor molecule, the apoptosis-associated speck-like protein containing a CARD (ASC), and the effector protease caspase 1 (16). Moreover, the NLRP3 inflammasome includes three crucial regions (**Figure 1**): i) a C-terminal leucine-rich repeat domain (LRR), that recognizes and binds both pathogens and danger associated molecular patterns (PAMPs and DAMPs, respectively); ii) a central triple-ATPase domain called NACHT, responsible for mediating nucleic acid ligation as well as protein oligomerization and iii) a N-terminal effector domain consisting of a caspase recruitment domain (CARD) or a pyrin domain (PYD), to which intracellular signaling pathways downstream transmission is attributed.

The PYD and CARD domains form the adaptor protein ASC, which promote the binding of NLRP3 and caspase-1; pro-caspase-1 is the precursor molecule of the effector protein caspase-1, which gives rise to its active form through self-cleavage and promotes the maturation and release of pro-inflammatory cytokines, among the most important, IL-1β and IL-18 (17).

### Activation mechanisms

The exact molecular signal leading to activation of the NLRP3 inflammasome remains to be clarified but currently several activation mechanisms, not mutually exclusive, have been proposed and include: i) ionic flux, ii) mitochondrial dysfunction with production of reactive oxygen species (ROS) and iii) lysosomal damage (18).

**Figure 2:**
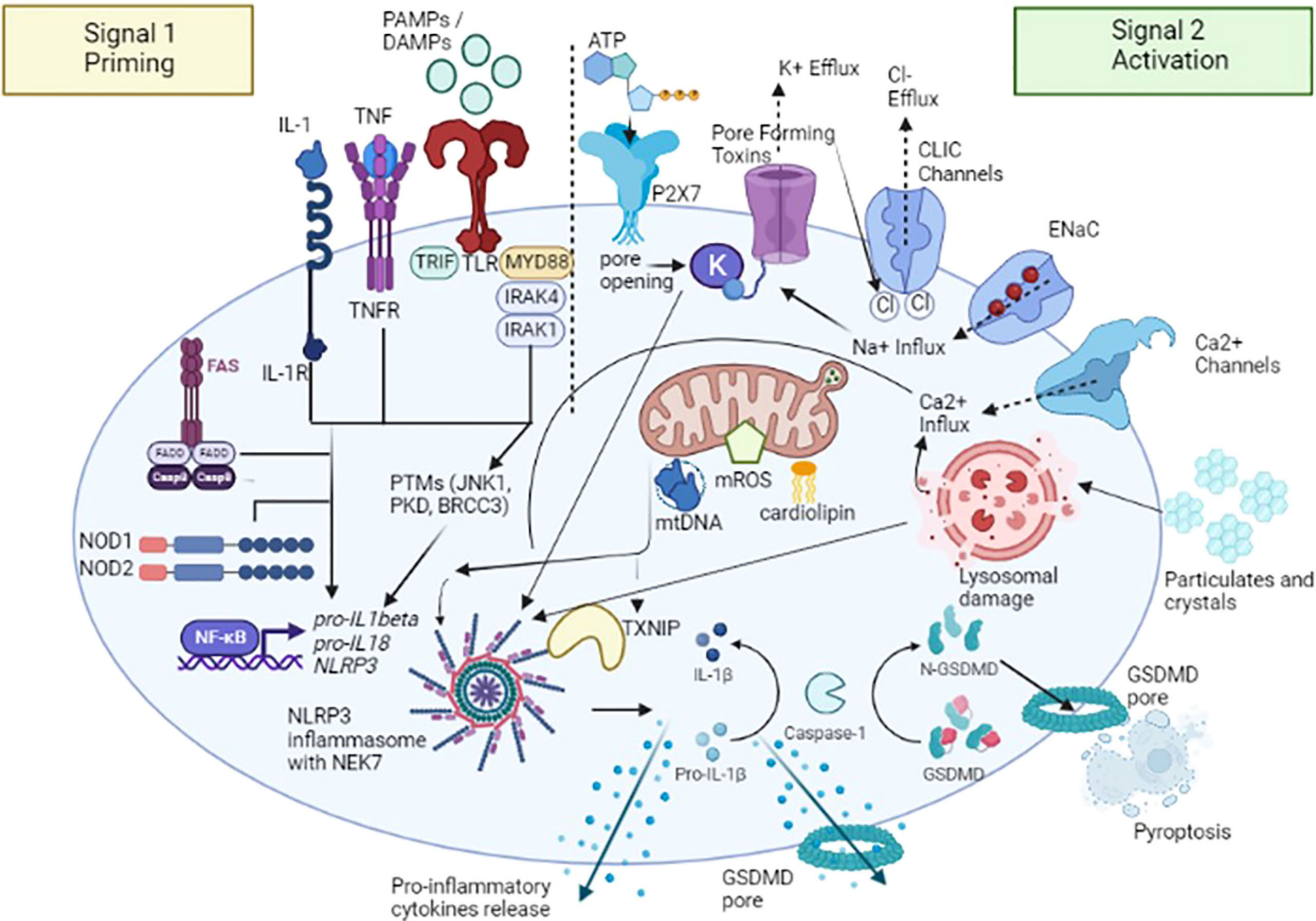
A two-signal model for NLRP3 inflammasome activation. The signal 1 – priming – (on the left) is provided by the binding between pathogen-associated molecular patterns (PAMPs) and DAMPs (damage-associated molecular patterns) with toll-like receptors (TLRs), tumor necrosis factor (TNF) and its receptor (TNFR) and IL-1β and its receptor (IL-1R), promoting the activation of the transcription factor NF-κB and subsequent upregulation of NLRP3, pro-interleukin (IL) -1β, and pro-IL-18. Caspase-8 and FAS-mediated death domain protein (FADD), and NOD (nucleotide oligomerization domain) 1/2 are also involved in the priming step by regulating the NF-κB pathway. NLRP3 undergoes post-translational modifications that allow its activation. The signal 2 – activation - (on the right) is provided by several stimuli including extracellular ATP after binding with purinergic receptor type 2, family X, subunit 7 (P2X7) channel, pore-forming toxins and specific particulates and crystals. Three main NLRP3 activation mechanisms have been proposed: i) ion redistribution model including potassium (K+) efflux, calcium (Ca2+) mobilization and sodium (Na+) influx and chlorine (Cl−) efflux, ii) mitochondrial dysfunction with the release of mitochondrial ROS (mROS), mitochondrial DNA (mtDNA), and cardiolipin and iii) lysosomal damage. NLRP3 activation also requires NIMA-related kinase 7 (NEK7), which binds to the NLRP3 leucine-rich repeats (LRRs) and is required for its oligomerization. Activated caspase-1 cleaves the gasdermin D (GSDMD), an effector protein of pyroptosis, forming transmembrane pores that facilitate the release of mature IL-1β, IL-18 and other pro-inflammatory cytokines. TXNIP, NLRP3 ligand mitochondria/thioredoxin-interacting protein; CLIC, Chloride intracellular channel; ENaC, Epithelial sodium channel. Figure has been created with BioRender.com.

NLRP3 inflammasome activation constitutes the two-part hypothesis (**Figure 2**). Briefly, “Signal 1” occurs when PAMPs and DAMPs prime the cell through toll-like receptors (TLRs), as well as after binding between tumor necrosis factor (TNF) and its receptor (TNFR) and IL-1β and its receptor (IL-1R), located on the cell membrane surface, so that nuclear factor-κB (NF-κB) signaling pathway activated can upregulate, at the transcription level, the expression of the NLRP3 oligomers pro-IL-1β and pro- IL-18 (19). Moreover, both signaling molecules MyD88 (Myeloid differentiation primary response 88) and TRIF (TIR-domain-containing adapter-inducing interferon-β) of the NF-κB signaling pathway regulate the induction of NLRP3 and pro-IL-1β in response to TLR ligands (20). Some studies revealed that both apoptotic signaling molecules caspase-8 and FADD (FAS-associated death domain) are also required for NLRP3 induction during the priming step (21, 22), as well as NOD1/2 (20). “Signal 2” consists of the NLRP3 activation to promote the assembly of multi-protein complexes; after PAMPs or DAMPs recognition through the C-terminal LRR domain, the PYD (NLRP3) -PYD (ASC) interactions form a protein complex. Subsequently, ASC promotes the recruitment of pro-caspase-1, through CARD domain, to activate the effector protein; once activated thorough its proteolytic hydrolysis, caspase-1 cleaves pro-IL-1β and pro-IL-18 and promotes the maturation and release of IL-1β and IL-18, thus triggering a downstream inflammatory response (23). Activated caspase-1 also cleaves the gasdermin D (GSDMD), an effector protein of pyroptosis, forming transmembrane pores that facilitate the release of mature IL-1β and IL-18 (24) and interfere with the ion and water flux, thus resulting in a strong inflammatory state and cell death. This pathway is currently designated as “canonical NLRP3 inflammasome activation” (25).

As mentioned above, NLRP3 can be activated by a wide spectrum of stimuli following the priming step, including extracellular adenosine triphosphate (ATP), potassium (K+) efflux (26), medically relevant crystals such as crystalline silica (27), pathogen-associated RNA - mRNA, tRNA, and rRNAs - (28), and bacterial, viral and fungal components (29, 30). NLRP3 doesn’t seem to interact directly with any of these agonists and it is speculated that they induce a common cellular signal (31). Currently, it is considered that the activation pathways of the NLRP3 inflammasome mainly include three mechanisms; the first is represented by ion efflux, in particular K+ efflux; under the stimulation of extracellular ATP, K+ ion redistribution is activated by the purinergic receptor type 2, family X, subunit 7 (P2X7) channel and pannexin-1 pore, thus causing the activation of NLRP3 inflammasome (32). New insights were provided by the identification of NIMA-related kinase 7 (NEK7), an NLRP3-binding protein that acts downstream of K+ efflux to regulate NLRP3 oligomerization and activation. In the absence of NEK7, caspase-1 activation as well as IL-1β release were abolished in response to signals that activate NLRP3 (33). On the other hands, a recent study demonstrated that NEK7 is insufficient to induce activation, but it could facilitate NLRP3 oligomerization by bridging *NLRP3* protomers (34). In addition to potassium, both calcium (Ca2+) mobilization and sodium/chlorine (Na+/Cl-) redistribution, specifically Na+ Influx and Cl− Efflux, seem to play an important role in the NLRP3 activation (4). The second mechanism proposed is the generation of ROS: after stimulation, the products derived from mitochondria and other mitochondrial signaling molecules, such as mitochondrial ROS (mROS), mitochondrial DNA (mtDNA), and cardiolipin, contribute to the NLRP3 inflammasome activation (35). It has been proposed that the overexpression of mROS could activate the NLRP3 inflammasome through mainly two-signal models, NF-κB signaling pathway and NLRP3 ligand mitochondria/thioredoxin-interacting protein (TXNIP). This latter is activated upon ROS accumulation; the binding of circulating TXNIP to NLRP3 protein seems to promote its activation (36), even though recent studies showed that this process is involved more in the sensitization of NLRP3 than in its activation (37). The third NLRP3 activation mechanism proposed is the lysosome damage: crystals or protein aggregates can induce lysosomal swelling, leakage, and release of proteases such as cathepsin B or promote cell death, thus generating further inflammasome stimuli (38).

Alterations in NLRP3 inflammasome activation process can lead to exacerbated inflammatory loop and tissue damage, highlighting the importance of selective triggers and closed regulation of inflammasomes. Several regulatory mechanisms, including transcriptional and post-translational modifications (PTMs) of sensor proteins as well as other key inflammasome-associated proteins have been identified to regulate NLRP3 inflammasome activation (39). Of these modifications, the phosphorylation and ubiquitination are the best characterized (**Figure 1**); for example, NLRP3 can be phosphorylated at specific residues, such as phosphorylation at Ser5 (40) and Tyr861 residues (41) in the PYD and LRR domains, respectively, promoting the inhibition of NLRP3 activation, preventing both its homo-oligomerization and interaction with ASC. Notably, AKT kinase is the enzyme responsible to phosphorylation at Ser5 residue (42), while the protein phosphatase 2 (PP2A) can reverse this process by dephosphorylating Ser5 domain of NLRP3, thus disrupting the interaction between NLRP3 and ASC (40). Similarly, the tyrosine-protein phosphatase non-receptor type 22 (PTPN22) has been shown to directly interact with NLRP3 and dephosphorylates Tyr861 residue, leading to NLRP3 activation (43). In contrast, phosphorylation at Ser198 residue, mediated by Jun N-terminal kinase-1 (JNK1), promotes inflammasome activation and it has been reported to be an essential priming step and required for NLRP3 self-association (44). Interestingly, the blockade of Ser198 phosphorylation by mutating Ser198Ala or inhibiting JNK1 in a CAPS mouse model, prevents the Lipopolysaccharide (LPS)-induced CAPS-associated NLRP3 activation, confirming that phosphorylation at Ser198 residue constitutes an essential priming event for NLRP3 inflammasome activation and suggesting that JNK1 inhibition could be considered a promising potential target for CAPS or other NLRP3-related diseases (44). Another study conducted by Zhang et al. (45) showed that phosphorylation at Ser 295 residue mediated by the Golgi-associated protein kinase D (PKD) recruits NLRP3 from mitochondria-associated endoplasmic reticulum membranes (MAMs) resulting in assembly of the active inflammasome. Moreover, the same authors demonstrated that PKD inhibition of LPS-stimulated peripheral blood mononuclear cells (PBMCs) isolated from CAPS patients carrying the mutations.

NM-001079821.3: c.1307C>A, p.(Thr436Asn) or c.778C>T, p.(Arg260Trp) in the *NLRP3* gene resulted in a strong reduction of caspase-1 cleavage and IL-1β secretion as compared with control-treated cells; these findings corroborated that PKD inhibition is sufficient to block inflammasome activity in cells of these patients with gain-of-functions mutations in the NLRP3 inflammasome (45).

Ubiquitylation also regulates NLRP3 activation by controlling both its degradation rate and ability to self-associate; F-box only protein 3 (FBXO3), Tripartite Motif Containing 31 (TRIM31) and Membrane Associated Ring-CH-type finger 7 (MARCH7), triggered by TLRs, cause ubiquitylation of NLRP3 with a specific residue linkage type, resulting in its degradation (46, 47). On the other hand, BRCA1/BRCA2-Containing Complex Subunit 3 (BRCC3) (48), ubiquitin carboxyl-terminal hydrolase 7 (USP7) and ubiquitin carboxyl-terminal hydrolase 47 (USP47) deubiquitylates NLRP3, enabling its homo-­oligomerization (49). Other PTMs, including nitrosylation, SUMOylation, acetylation, and ADP-ribosylation have been found to be involved in the regulation of NLRP3 inflammasome activation, as extensively reviewed by Zangiabadi et al. (50).

Finally, there are also several endogenous cytokine receptor inhibitors, including IL-1 receptor antagonist (IL-1RA) and IL-18 binding protein that, through the binding to their receptor, prevent downstream inflammation with the blockage of cytokines release (51).

## The CAPS spectrum: Clinical, laboratory and genetic findings

CAPS encompass a spectrum of three overlapping autosomal dominant (AD) autoinflammatory disorders caused by heterozygous gain-of-functions mutations in *NLRP3* gene: familial cold autoinflammatory syndrome (FCAS, OMIM 120100), Muckle-Wells syndrome (MWS, OMIM 191900) and chronic infantile neurologic, cutaneous and articular syndrome/neonatal-onset multisystem inflammatory disease (CINCA/NOMID, OMIM 607115) (**Table 1**). These disorders are clinically characterized by recurrent episodes of fever, urticaria-like neutrophilic rash, joint pain and systemic “flu-like” inflammatory symptoms associated to variable organ involvement depicting a clinical spectrum in which FCAS represents the mildest phenotype with less severe fever attacks, mainly dependent on cold exposure, MWS is considered as an intermediate phenotype in which the inflammatory attacks, although less dependent on cold exposure, last longer and could be complicated with late-onset amyloidosis, and CINCA/NOMID at the end of the spectrum characterized by continuous flares associated to severe central nervous system (CNS) and arthropathic involvement leading to a poor life expectancy and severe sequelae (52).The true prevalence of CAPS is not well defined yet, ranging from 2.7 to 5.5 per 1 million and might be higher, as CAPS could often being misdiagnose (53, 54). The distribution of different CAPS phenotypes varies around the world; up to 75% of CAPS patients in North America are diagnosed as FCAS, due to a founder mutation (p.Leu353Pro) in *NLRP3* gene, MWS represents the most common phenotype reported in Europe, while CINCA/NOMID patients are less common since the majority of cases are caused by *de novo NLRP3* mutations (12) (**Table 2**).

Based on pathogenetic landscape, in a recent consensus proposal, it has been proposed the term “NLRP3-associated autoinflammatory diseases (NLRP3-AID)” for the CAPS spectrum (55), but it seems that this new taxonomy failed to reach a global consensus, preferring the term CAPS.

### Familial cold autoinflammatory syndrome (FCAS)

FCAS was first described in 1940 by Kile and Rusk, in a proband with recurrent fever, urticarial eruption, chills, and joint swellings following cold exposure, with a positive family history for these symptoms, defining this entity as familial cold urticaria (56). Later on, more than ten FCAS pedigree have been described, mainly presenting with early-onset, short-living (6 to 10 hours) attacks consisting of fever, urticaria-like eruption, chills and arthralgias following cold exposure, in particular cold air (57); urticaria-like skin biopsies showed a predominance of neutrophils in early-stage lesions while lymphocytes in long-standing ones (57). In 2000, through a genetic linkage analysis of five affected families, Hoffman and colleagues identified a common locus on chromosome 1q44 for both FCAS and MWS (58), and one year later, the same authors discovered four *NLRP3* causative variants of both these phenotypes (59). FCAS clinical diagnostic criteria are represented by early-onset (<6 months), recurrent-intermittent episodes of fever and rash that follow natural or experimental cold exposure, usually lasting less than 24 hours and accompanied by conjunctivitis and absence of serositis; sensorineural deafness and lymphadenopathy have also been reported, although the latter symptoms are less specific for FCAS and could be present in other CAPS subtypes, highlighting the evidence of a clinical overlap between these phenotypes (60). The symptoms usually start 2 hours after cold exposure, first with the appearance of an urticarial rash and then progress at 4-6 hours with fever and arthralgias; nausea, vomiting and thirst are frequent while sensorineural hearing loss and amyloidosis are rarely seen, with a life expectancy comparable to that of the general population (60). Laboratory examinations fail to be pathognomonic; in some patients an increase of blood neutrophilia and interleukin (IL)-6 is observed during the flares, while C-reactive protein (CRP) and Erythrocyte Sedimentation Rate (ERS) could be slightly elevated at baseline and did not significantly increase during the attacks (12). The main differential diagnoses of FCAS include isolated acquired cold urticaria, in which family history is negative and no systemic symptoms are observed during the attacks, and the newly described autosomal dominant forms of familial cold urticaria due to alterations in *NLRP12* (61), *PLCG2* (PLCG2-associated antibody deficiency and immune dysregulation - PLAID syndrome, in which urticaria-like rash is associated to antibody deficiency) (62), and *NLRC4* genes (63). The latter findings support the crucial role of genetic testing to achieve an appropriate diagnosis in recurrent urticaria-like rash associated to fever and systemic symptoms during the infancy.

### Muckle-wells syndrome (MWS)

MWS syndrome represents the intermediate phenotype of CAPS spectrum and is clinically characterized by early-onset episodic fever and skin rashes, lasting for 1-to-3 days, progressive sensorineural hearing loss and late-onset amyloidosis (52). Muckle and Wells first described in 1962, a five-generation family affected by urticaria, progressive sensorineural hearing loss and renal amyloidosis (64). Some years later, Muckle summarized the clinical features in 78 MWS cases as following: the urticaria-like skin rash – more aching than pruritic- accompanied by fever and arthralgias as constant findings, whereas sensorineural hearing loss was present in more than half of patients and only one third of the cases presented amyloidosis (65). In 1999, Cuisset et al., through a genome-wide study, in three affected families identified a locus at chromosome 1q44 responsible for MWS (66), while, as mentioned above, in 2011 Hoffman et al., identified *NLRP3* pathogenic variants associated with both MWS and FCAS (59). Although progressive sensorineural hearing loss and the tendency to develop renal amyloidosis, with the increase of serum levels of amyloid A protein (SAA), are considered the most specific findings in MWS, atypical symptoms such as severe abdominal pain, recurrent genital and oral ulcers, and livedo reticularis have also been reported in some families (67, 68), together with the widely reported MWS/FCAS (69–73) and MWS/CINCA/NOMID (74–76) overlap features.

### Chronic infantile neurologic, cutaneous and articular syndrome/neonatal onset multisystem inflammatory syndrome (CINCA/NOMID)

CINCA/NOMID represents the most severe phenotype of the CAPS spectrum and is clinically characterized by an early-onset, recurrent-to-continuous urticaria-like rashes associated to fever, higher levels of acute phase reactants and severe articular and neurological involvement (77). Prieur and Griscelli, first described this syndrome in 3 patients presenting with an unusual clinical picture characterized by persistent maculo-papular rash, polyarthritis, papilledema, and chronic neutrophilic meningitis (78), coining the term CINCA to describe this specific entity, occurring both *de novo* and in familial cases (79). In United States literature, the syndrome was first described by Lorber et al. and termed NOMID, in which peculiar epiphyseal radiographic signs were found, subsequently described also by Torbiak et al. in a series of 31 patients (80). In 2002, Feldmann and colleagues identified *NLRP3* pathogenic variants associated to CINCA/NOMID; none of these pathogenic variants were previously described in FCAS and MWS family, and the majority of probands presented *de novo* mutations (81). In the same year, in a similar cohort of 6 patients, Aksentijevich *et al*, independently confirmed the association between *NLRP3* genetic changes and these phenotypes, thus confirming that FCAS, MWS and CINCA/NOMID were all caused by *NLRP3* pathogenic variants (82). CINCA/NOMID patients present with congenital, usually persistent, urticaria-like rash accompanied by neutrophilic leucocytosis and high level of CRP and showing typical facies characterized by frontal bossing, saddle-back nose and large cephalic perimeter (83). The most common neurological manifestations include chronic aseptic meningitis, headache, intellectual disability, hydrocephalus, and seizures, all of which could be life-threating and/or lead to severe sequelae (83–85). Ocular involvement is frequent, with high rates of anterior uveitis, chronic papilledema and optic nerve atrophy which could lead to visual loss if untreated (69, 86); it is estimated that nearly half of patients develop early-onset sensorineural hearing loss due to persistent cochlear inflammation (83, 87). Severe arthropathy is a leading symptom of this disease and derives from disorganized bony overgrowth due to abnormal endochondral bone formation (88); it mainly involves the knees, but elbows, wrists and ankles could also be affected (88). Early degenerative arthropathy and joint contractures represent the most common sequelae (83). Amyloidosis in CINCA/NOMID is not so far reported in literature, possibly due to the high mortality of this condition in early infancy before the introduction of targeted therapies, which could nowadays prevent the long-term complications (73). Due to the severe systemic involvement, CINCA/NOMID life expectancy is low, sequelae are frequent and highly affect patients’ quality of life.

### CAPS-causing mutations in *NLRP3* inflammasome

In 2001, heterozygous causative variants in the *NLRP3* gene have been identified in FCAS and MWS patients and subsequently in CINCA/NOMID syndrome (12). Today, more than 100 *NLRP3* variants are classified as pathogenic/likely pathogenic with strong genotype-phenotype correlation along the disease continuum (89). Most of these variants are missense substitutions mainly concentrated in exon 3 which encodes for the central NACHT domain (89); some pathogenic variants affecting C-terminal exons encoding for the LRR domain have been also found, as in the recent work of Vahedi et al., in which the authors described a new variant in exon 5, coding for the LRR domain, of the *NLRP3* gene (NM-001079821.3: c. 1060 G>T, p.(Ala354Ser) (90). All these CAPS associated *NLRP3* pathogenic variants are gain of function leading to an hyperactivation of NLRP3 inflammasome, an increased secretion and release of pro-inflammatory cytokines and tissue damage related to disease symptoms. Current evidence suggests that the wild-type NLRP3 is maintained in an inactive state through auto-inhibition mechanisms mediated by the interaction between the LRR domains with the NACHT domain of the protein; according to this model, pathogenic variants in the NACHT domain and/or C-terminal regions encoding for the LRR domain could, thus, alter this auto-inhibitory mechanism, leading to an increased inflammasome activation and subsequent IL-1β release. This physio-pathological scenario has been validated trough different studies (**Table 3**), helping to increasingly untangle the complex biology of NLRP3 in health and disease. In an *ex vivo* study conducted by Agostini *et al*, monocytes isolated from a Muckle-Wells patient carrying NM_001243133.2(NLRP3):c.778C>T, p.(Arg260Trp) mutation, have been stimulated with LPS without ATP; these cells displayed a spontaneous increase of ASC speck formation, caspase-1 cleavage, IL-1β release, and pyroptosis, despite the absence of an activation signal, normally required for NLRP3 inflammasome assembly (91).

**Table 3:** Main genetic functional studies of CAPS-causing mutations.

Human mutation	Mouse mutation	Human phenotype	Experimental approach	Conditions of activation	Effect of mutation	Author, year/Reference number
NLRP3:c.778C>T; p.(**Arg260Trp**)	.	MWS	1.Monocytes isolation and purification2. ELISA and Western Blot using antibody specifically directed against the active fragment (p17) of proIL-1β	LPS	Spontaneous secretion of active IL-1β	Agostini et al., 2004/^91^
NLRP3:c.778C>T, p.(**Arg260Trp**); c.907G>A, p.(**Asp303Asn**); c.1880A>G, p.(**Glu627Gly**)	.	MWS, CINCA/NOMID, FCAS	1. Monocytic THP-1 cell line expressing mutant proteins2. NF-kB Assay3. IL-1 ELISA4. Immunoprecipitation5. Small Interfering RNA	ASC-dependent	1.Constitutive activation of NLRP32. Induction of NF-kB activation3. Increased IL-1β secretion4. Increased ASC binding	Dowds et al., 2004/^94^
NM_001243133.2(NLRP3):c.1058T>C,p.(**Leu353Pro**); c.1687T>A, p.(**Tyr563Asn**); c.1976T>A, p.(**Met659Lys**) and c.1573G>A, p.(**Glu525Lys**)/c.592G>A, p.(**Val198Met**),	.	FCAS	1.Adherence-enriched monocytes2. quantitative PCR and ELISA	Cold exposure (32°C and 37°C)	1. Increased cytokine transcription at 16 hours with 32°C of incubation.2.Response to mild hypothermia with an increased IL-1β release and secretion of IL-6 and TNF-α	Rosengren et al., 2007/^96^
NM_001243133.2(NLRP3):c.1058T>C,p.(**Leu353Pro**); c.1687T>A, p.(**Tyr563Asn**)	.	FCAS	1.Cell lines (HeLa cells) expressing fusion proteins2. IL-1β secretion assay3. Western-blot analysis4. Reverse transcription and real-time PCR5. Ca2+ imaging	Cold exposure	1.Inflammasome assembly Ca2+- mediated2. Cryo-sensitive foci formation	Karasawa et al., 2022/^97^
NM_001243133.2(NLRP3):c.907G>A, p.(**Asp303Asn**); c.1709A>G, p.(**Tyr570Cys**)	.	CINCA/NOMID	1.Cell lines (HeLa cells) expressing fusion proteins2. IL-1β secretion assay3. Western-blot analysis4. Reverse transcription and real-time PCR5. Ca2+ imaging	Cold exposure	1.Inflammasome assembly Ca2+- mediated2. Cryo-sensitive foci formation	Karasawa et al., 2022/^97^
NM_001243133.2(NLRP3):c.1058T>C; **p.Leu353Pro**	p.Leu351Pro	FCAS	1.Conditional knockin;Nlrp3A350VneoR/+ and Nlrp3L351PneoR/+ mice2. *In vitro* studies: myeloid cells isolation	1.LPS2.Cold exposure	Hyperactivation of the NLRP3	Brydges et al., 2009/^98^
NLRP3:c.778C>T; p.(**Arg260Trp**)	p.Arg258Trp	MWS	Constitutive knockin	LPS	1.Increased IL-1β and IL-18 production from APCs2. Contextual increase of Th17 differentitation	Meng et al., 2009/^99^
NLRP3:c.907G>A; p.(Asp303Asn	.	CINCA/NOMID,MWS	1. C57BL/6J mice2. HEK293 cells transfection2.Isolation of particles of recombinant ASC and NLRP33.Coimmunoprecipitation and immunoblot analysis4. ELISA5. Multiplex flow cytometry	1.LPS2.Extracellular caspase-13.Sorrounding macrophage	Increased extracellular release of functional oligomeric inflammasome particles containing both NLRP3 and ASC	Baroja-Mazo et al.,2014/^100^
NLRP3:c.907G>A; p.(**Asp303Asn**)	p.Asp301Asn	CINCA/ NOMID	Conditional knockin; Nlrp3fl(D301N)/+ mouse models; Gsdmd−/− mice	LPS	1.Constitutive maturation of GSDMD2. Excessive pore formation3. Increased and constitutive production ofIL-1β	Xiao et al., 2018/^101^

CAPS, Cryopyrin associated periodic syndromes; MWS, Muckle Wells Syndrome; LPS, Lipopolysaccharide; ATP, adenosine triphosphate; ASC, apoptosis-associated speck-like protein containing a CARD; IL, interleukin; GSDMD, gasdermin D; CINCA/NOMID, chronic infantile neurologic, cutaneous, and articular syndrome/neonatal-onset multisystem inflammatory disease; FCAS, Familial cold autoinflammatory syndrome; Th, T-helper.

PCR, Polymerase Chain Reaction; ELISA, Enzyme-linked immunosorbent assay; Th17, T helper 17 cells; APCs, Antigen-presenting cells.

In 2004, Neven et al., based on the discovery of seven new mutations and all pathogenic variants described at the time in the NACHT domain and its flanking regions, from which the probable existence of mutational hotspots, created a 3-dimensional model of the nucleotide-binding domain of NLRP3, showing that most of the mutations clustered in a region predicted to be involved in intermolecular contacts; these findings suggested that genetic defects affecting nucleotide binding and hydrolysis as well as protein oligomerization functions could be responsible of NLRP3 dysregulation as observed in CAPS (92).

Similarly, in 2006, a computer-generated model of the NLRP3 structure confirmed that NM_001243133.2(NLRP3):c.778C>T,p.(Arg260Trp) and c.907G>A, p.(Asp303Asn) mutations are localized within a region probably involved in oligomeric interactions, thus causing conformational changes in the oligomerization domain leading to increased self–self-interactions and oligomerization of NLRP3 (93).

After the first studies conducted by Agostini et al. (91), in order to characterize the molecular mechanisms of CAPS, Dowds et al., generated constructs to express three of the most common CAPS-causing mutations (NM_001243133.2(NLRP3): c.778C>T, p.Arg260Trp; c.907G>A, p.Asp303Asn and c.1880A>G, p.Glu627Gly), demonstrating that all mutant proteins induced strong ASC-mediated NF-κB activation compared to controls. Despite the absence of stimuli, CAPS-associated mutants, expressed in monocytic THP-1 cell line (which expressed endogenous ASC), induced a constitutive activation of NLRP3 promoted by an increased ability to interact with ASC, resulting in spontaneous secretion of IL-1β (94). Moreover, more recently, it has been demonstrated that the accelerated kinetics of IL-1β secretion by CAPS monocytes was caused by higher levels of ROS as well as an altered redox response to TLR triggers (95). Rosengren et al., in order to elucidate the cytokine response to cold exposure in monocytes isolated from FCAS subjects, incubated adherence-enriched monocytes at 32°C or 37°C; FCAS monocytes responded to 4h incubation at 32°C with significant secretion of IL-1β, while at 16h incubation also IL-6 and TNF-α were significantly elevated. Additionally, incubation at 32°C for about 1 hour was sufficient to induce an appreciable release of IL-1β, highlighting the important role of caspase-1 inhibitors in preventing the cold-induced early IL-1β release (96). FACS patients of this study harboured NM_001243133.2(NLRP3):c.1058T>C,p.(Leu353Pro); c.1687T>A, p.(Tyr563Asn); c.1976T>A, p.(Met659Lys) and c.1573G>A, p.(Glu525Lys)/c.592G>A, p.(Val198Met), not showing significant difference in cytokine release from monocytes (92). It is yet unclear why cold precipitates the attacks in FCAS, but a recent study showed that NLRP3 mutants (FCAS-associated NM_001243133.2(NLRP3):c.1058T>C,p.(Leu353Pro) and - c.1687T>A, p.Tyr563Asn, as well as CINCA/NOMID-associated NM_001243133.2(NLRP3):c.907G>A; p.(Asp303Asn) and - c.1709A>G, p.Tyr570Cys) formed cryo-sensitive, calcium-dependent molecular aggregates that could trigger inflammasome assembly, activation, and subsequent pro-inflammatory cytokine release (97).

To further explore the mechanisms involved in CAPS pathogenesis, engineered mutations causing FCAS and MWS have been assessed into mouse model by Brydges et al.; being Ala352 (c.1055C>T; p.Ala352Val) and Leu353 (c.1058T>C; p.Leu353Pro) residues (Ala350 and Leu351 in mouse NLRP3) highly conserved both in mouse and in human, Nlrp3A350VneoR/+ and Nlrp3L351PneoR/+ mice have been created. Bone marrow derived cells from these mice showed an hyperactivation of the NLRP3 inflammasome with cytokines release and speck formation with only the presence of LPS and/or exposure to cold temperature. Notably, these findings have been found only in cells from FCAS, but not MWS, or CINCA/NOMID mutant mice (98).

Almost simultaneously with these studies, Meng et al., analyzed the immune landscape of knock-in mice carrying a NLRP3 point mutation (p.Arg258Trp in mouse, p.Arg260Trp in human) MWS related, demonstrating that antigen presenting cells (APCs) from such mice produced great amounts of IL-1β and IL-18 upon stimulation with TLR ligands in the absence of ATP. In addition, these knock-in mice displayed a Th17-dominant response, demonstrating that this – and probably others - NLRP3 mutation leads to inflammasome hyperactivation supporting Th17 cell differentiation and tissue inflammation (99).

Baroja-Mazo et al. (100), first supported a model by which the NLRP3 inflammasome acted as an extracellular oligomeric complex, amplifying the inflammatory response; oligomeric inflammasome particles containing both NLRP3 and ASC, acting as danger signals, led to an amplification of the pro-inflammatory signals by promoting the activation of caspase-1 extracellularly and in surrounding macrophages after the particle’s internalization. These authors generated constitutively activated NLRP3 mutants (c.907G>A; p.(Asp303Asn) and through stimulation with caspase-1 extracellularly, as well as intracellularly after phagocytosis by neighboring macrophages, demonstrated the extracellular presence of oligomeric NLRP3 and ASC particles. These findings confirmed that inflammasome activation promoted the extracellular release of active inflammasome oligomers able to act as danger signals to amplify the inflammatory loop by activating caspase-1 (100).

Interestingly, CINCA/NOMID mouse models Nlrp3^fl(D301N)/+^, harbouring Asp301Asn mutation (ortholog of Asp303Asn in human NLRP3), demonstrated that Gasdermin D, a protein responsible of plasma membrane pores formation through which IL-1β and IL-18 could be secreted, is required for pyroptosis mediated to NLRP3 inflammasome, and its blockage could prevent the inflammatory pathway even in mice bearing *NRLP3* pathogenic variants; moreover, secretion of IL-1β and lactate dehydrogenase (LDH) was abolished in cells lacking GSDMD, suggesting that mature IL-1β was constitutively produced in CINCA/NOMID cells, but its release required GSDMD (101).

Somatic mosaicisms are also clinically relevant as it makes genetic diagnosis and counselling more challenging. In 2005, Saito et al., identified the first somatic mutation in a CINCA/NOMID proband, NM_001243133.2(NLRP3):c.1709A>G (p.Tyr570Cys) localized in the exon 3; the authors demonstrated that this somatic point variation increased the ability of NLRP3 to promote NF-kB activation in an ASC-dependent manner (102). Subsequently, Tanaka et al., identified somatic NLRP3 mutations in 69.2% (18/26) of mutation-negative CINCA/NOMID patients, estimating a level of mosaicism ranging from 4.2% to 35.8%; all these somatic mutations induced an increased ASC-dependent NF-κB activation (103). Overall, Labrousse et al. reported that the rate of CAPS-like patients carrying mosaicism ranged between 0.5% and 19%; up to now, 35 different somatic mutations have been reported in the *NLRP3* gene (104), giving rise to atypical phenotypes, milder disease course, or late-onset disease (105). Moreover, the genotype-phenotype correlation seems to be associated with the localization of the underlying mosaic variants; for example, the somatic mutation (p.Glu569Lys) in *NLRP3* gene identified by Louvrier et al., was localized in the NAD domain, in contrast to germline variant that was scattered in this latter. Functional studies demonstrated that this variant promoted higher ASC speck formation and IL-1β secretion when compared to controls. Moreover, all the germline mutations were associated with a severe phenotype, suggesting that mosaic state of this pathogenic variant could be incompatible with life if present in germinal state (106).

Due to the low frequency of the mutant allele, somatic mutations may not be detected using conventional methods of genetic analysis, such as Sanger sequencing, suggesting that next-generation sequencing (NGS) could be regarded as the gold standard diagnostic tool in CAPS patients, in order to formulate an appropriate diagnosis and leading to an early treatment and adequate follow-up.

Notably, also low penetrance variants in *NLRP3* gene have been reported in typical or atypical CAPS patients as well as in patients without apparent symptomatology, suggesting their role more in a genetic predisposition for common inflammatory disorders than in disease-causing alleles (107).

## Other *NLRP3-*related autoinflammatory diseases: deafness autosomal dominant 34 (DFN34) and keratitis fugax hereditaria (KFH)

DFN34 is a recently described NLRP3-related autoinflammatory disease (NLRP3-AID), clinically characterized by middle-age onset of bilateral, symmetric, and progressive sensorineural hearing loss (14); Nakashini and colleagues, first genetically characterized this syndrome in two unrelated families, showing a missense pathogenic mutation in the LRR domain of *NLRP3* gene (exon 7), NM_001243133.2(NLRP3): c.2753G > A; p.Arg918Gln (14). In the first family, hearing loss was accompanied by autoinflammatory signs and symptoms, while in the second family hearing loss segregated without any other organ involvement, suggesting that resident macrophage/monocyte-like cells in the cochlea could mediate local autoinflammation *via* NLRP3 inflammasome activation (14). Moreover, some of these patients presented elevated inflammatory markers (ESR, PCR) and an abnormal secretion of IL-1β by PBMCs in response to LPS stimulation, similar to that observed in CAPS. Magnetic resonance imaging (MRI) of the subjects showed cochlear pathologic signal enhancement, suggesting that a persistent autoinflammatory activation within the cochlea could be responsible for the hearing loss (14); These MRI findings as well as cochlear inflammation have been also reported in MWS and CINCA/NOMID, in which sensorineural hearing loss represents a distinctive symptom (77). A second pedigree worldwide of non-syndromic DFN34 has been described by Kim and colleagues (108), which identified a novel pathogenic missense variant in the exon 7, NM_001243133.2(NLRP3): c.2752G > A; p.Arg918X, located in the LRR domain and associated with a severe hearing status; this finding has been also supported by *in vitro* IL-1β assay, leading to the therapeutic choice of IL-1β antagonist which proved effective with a significant raising of the hearing threshold (108). Basing on these findings, it is still matter of debate if DFN34 should be considered a distinct entity related to *NLRP3* gain-of-function or a milder CAPS phenotype restricted to the inner ear.

Keratitis fugax hereditaria (KFH) is a monogenic, autosomal dominant, autoinflammatory syndrome with an exclusive eye involvement, up to date only reported in Finnish population (109). KFH phenotype was first described in 1964 by Valle, in a Finnish family with 10 members affected (110), and later it has been termed kerato-endothelitis fugax hereditaria due to presumptive corneal endothelial abnormalities found in 21 affected patients in another Finnish family (111). In 2018, Turunen and colleagues detected, in 34 KFH patients (30/34 belonging to 7 affected families and 4/34 sporadic cases), the following pathogenic variant in exon 1 of *NLRP3* gene, NM_001243133.2(NLRP3): c.61G > A, that leads to substitution of histidine for aspartic acid at position 21 (p.Asp21His) in the pyrin domain, thus expanding the spectrum of NLRP3-AIDs (15). KFH clinically presents with unilateral and recurrent attacks of conjunctival and peri-corneal injection, pain, and photophobia, usually starting around the second decade of life, lasting 24-48 hours, and resolving without significant sequelae apart from blurry vision some weeks after the acute attack and reduced visual acuity in the long-term period; the attacks have been reported to decrease in frequency and intensity during lifetime (15). Ultrastructural studies with *in vivo* corneal confocal microscopy (IVCM) have demonstrated that the primary autoinflammatory site is the middle and anterior stromal layers of the cornea, and since the endothelium is not actually involved, the term keratoendotheliitis should be avoided as misleading; moreover, recurrent attacks cause stromal opacities possibly due to extracellular debris and lipid deposition which could lead to reduced visual acuity (112). No associated systemic inflammatory symptoms have been associated to KFH in the Finnish cohort, and none of the ocular symptoms occasionally described in CAPS seems to overlap with KFH findings, suggesting that KFH could be an NLRP3 eye-restricted autoinflammatory disease (113). Though a founder effect for the pathogenic variant has been established considering the clustering of the disease, it is also true that only a high index of suspicion together with the appropriate genetic testing could assure the correct diagnosis of KFH, which could be misdiagnosed as anterior uveitis or herpetic keratitis due to the self-limited course (113). Considering that KFH is a newly characterized NLRP3-AID, many cases that could have been previously undiagnosed may be now correctly identified also in non-Finnish patients, to further elucidate the clinical and physio pathological scenario of this intriguing eye-specific autoinflammatory disease.

## NLRP3 inflammasome-directed therapies for the treatment of CAPS and beyond

The discovery of the crucial role of NLRP3 inflammasome pathway hyperactivation with the subsequent IL-1β overexpression in the pathogenesis of CAPS, has led to consider NLRP3 inflammasome and IL-1 β blockade a promising and effective tailored treatment for this disease. During the last years, IL-1β cascade inhibitors - anakinra, canakinumab, rilonacept, - the latter only approved in the US - have been shown to be effective and relatively safe, representing a breakthrough for the therapeutic management of CAPS, which are now currently treatable in both the acute phase than in the long-term maintenance (114, 115) (**Table 4**). Isolated reports and *in vitro* studies suggested anti-TNF-α agents as a valuable therapeutic option in CAPS, although they appear inferior compared to IL-1 antagonists in symptoms relief and disease control (116, 117) while IL-18 inhibition, a key cytokine released upon inflammasome activation, although promising did not show positive results in preclinical and clinical studies, and currently no FDA approved IL-18 antagonist is available in the clinical practice (118). Due to the pivotal role of the NLRP3 inflammasome in a wide array of autoinflammatory and chronic inflammatory diseases, pharmacologic research of NLRP3 cascade inhibitors is actively growing and many compounds targeting NLRP3 inflammasome and its related pathway are currently investigated, as extensively reviewed by Zahid et al. (119).

**Table 4:** Principal clinical trials involving IL-1 antagonists for CAPS patients.

Author, year/reference number	Study type, NCT number	Drug(s)	Details of treatment	Number of patients, clinical phenotype	Study duration	Main study results
Goldbach-Mansky et al., 2006 (126)	Open label, NCT00069329	Anakinra	1-to-2- mg/kg/day	18, CINCA/NOMID	6 months	Clinical remission in all patients Reduction of SAA, CRP, ESR serum level Improvement of CNS abnormalities, shown by MRI
Sibley et al., 2012 (121)	Open label, NCT00069329	Anakinra	1-to-5 mg/kg/day	26, CINCA/NOMID	60 months	Improvement in patients’ and physicians’ disease scores Reduction of systemic inflammatory markers Significant decrease in CNS inflammation
Goldbach-Mansky et al., 2008 (120)	Open label, NCT00094900	Rilonacept	300 mg (induction dose) 100-to-360 mg/week (maintenance dose)	5, FCAS	24 months	Clinical remission and decrease in inflammatory markers
Hoffman et al.,2012 (73)	Open label, NCT00288704	Rilonacept	160 mg/week (adults) 2.2 mg/kg (paediatrics)	101, - 95 FCAS - 3 MWS - 3 FCAS/MWS overlap	18 months	Improvement of clinical sign and symptoms, and normalized inflammatory markers
Lachmann et al., 2009 (76)	RCT, NCT00465985	Canakinumab, placebo	150 mg every 8 weeks	35, - 34 MWS - 1 MWS/CINCA/NOMID overlap	6 months	90% clinical remission in treatment group Normal levels of SAA, CRP in treatment group
Kuemmerle-Deschner et al., 2011 (131)	Open label, NCT00685373	Canakinumab,	150 mg every 8 weeks	166, - 30 FCAS - 103 MWS - 32 CINCA/NOMID	24 months	Complete remission in 78% canakinumab-naive patients Improvement in clinical and laboratory parameters in the entire cohort

CINCA/NOMID, Chronic infantile neurologic, cutaneous, and articular syndrome/neonatal-onset multisystem inflammatory disease; SAA, serum amyloid A protein; CRP, C-reactive protein; ESR, erythrocyte sedimentation rate; CNS, central nervous system; MWS, Muckle Wells Syndrome**;** FCAS, Familial cold autoinflammatory syndrome; RCT, Randomized controlled study.

### Anakinra (IL-1 receptor antagonist)

Anakinra is the first recombinant human selective IL-1 receptor antagonist developed, that acts blocking both IL-1β and IL-1α activity; it has a half-life of 4 to 6 hours, and subcutaneous daily administration is required (114).

In 2006, Goldbach-Mansky et al., performed the first non-randomized clinical trial in 18 CINCA/NOMID patients with anakinra at the dosage of 1-2 mg/kg per day for 6 months; all the patients reported a substantial improvement in clinical symptoms, including urticaria-like rash, conjunctivitis, hearing loss and CNS inflammation, as demonstrated by MRI imaging (120). Moreover, anakinra downregulated systemic inflammation as serum levels of IL-6, TNF and IL-1β decreased during the therapy; flare-ups have been observed during the withdrawal period and responded to drug re-injection (120). A 3-to-5-year follow-up study in 24 CINCA/NOMID patients treated with anakinra showed the sustained remission in most of the subjects, together with a safety profile, the improvement of physician and patients’ quality of life assessment, as well as the normalization of systemic and organ inflammation, with the exception of bone involvement which did not respond to the therapy (121). Observational studies in different cohorts of patients confirmed these results with a complete and partial remission rate ranging from 40% up to 100% among different studies (70, 114, 122, 123). In conclusion, anakinra shows a good safety profile, also in pregnancy (124), and could prevent and stabilize systemic and organ-specific autoinflammatory damages, including hearing loss in DFN34 patients (108, 114).

### Rilonacept (IL-1 trap)

Rilonacept is an FDA approved dimeric fusion protein consisting of the extracellular binding portion of IL-1 receptor and the IL-1 accessory protein linked to the Fc portion of the human IgG1, capable to bind and inactivate circulating IL-1β and IL-1α; it has a half-life of around one week and it is scheduled as weekly subcutaneous injections (125). Rilonacept was first used in a pilot study by Goldbach-Mansky to treat 5 FCAS patients, showing a rapid and excellent clinical and biochemical efficacy, with a good safety profile and a sustained response up to two years (126).

A subsequent 24-weeks randomized clinical trial followed by an open-label extension up to 72 weeks in patients with FCAS and MWS showed that rilonacept reduced the number of flares and symptoms in 84% of treated patients compared to placebo, and significant improved patient’s and physician global assessments, SAA and CRP serum levels, as well as limitations of daily activities scores, with a generally favourable and safety profile during the whole treatment period (, 74, 127). Despite its proved efficacy in the clinical trials, long-term safety concerns and the efficacy and availability of the most widely used IL-1 antagonist anakinra and canakinumab led clinicians to gradually move towards the latter for the treatment of CAPS (125).

### Canakinumab (anti IL-1β monoclonal antibody)

Canakinumab is a fully human IgG1 monoclonal antibody against circulating IL-1β, the main pro-inflammatory cytokine released from NLRP3 inflammasome; it has a long half-life (around 26 days) and could be dosed at 4- or 8-weeks intervals. One of the first multicentric, randomized, controlled clinical trial with canakinumab showed efficacy in 34/35 CAPS patients, leading to clinical remission starting from the first dose and maintained with subsequent administrations, both for clinical activity and inflammatory parameters (76); a study extension up to 48 weeks demonstrated a long-term remission, a decrease in inflammatory markers (SAA, CRP and IL-6), and the improvement of health-related quality of life (128). Comparable results were obtained in a Japanese cohort (100, 129). Another study in 6 CINCA/NOMID patients with a dose escalation regimen, demonstrated an improvement of clinical symptoms and inflammatory markers, although complete clinical remission was not achieved, especially for a persistent low-grade CNS leukocytosis (130). The largest cohort study of 166 CAPS patients with different phenotypes, both pediatrics and adults, showed that canakinumab led to a persistent clinical remission in 78% of the subjects, with no organ-damage progression, and a significant improvement in patients ‘quality of life (131).

Real-life observational studies in multiple cohorts confirmed the safety and the efficacy of canakinumab to induce a fast remission, long-term maintenance, and an efficacy on organ-specific outcomes such as hearing loss, migraine/headache, renal amyloidosis, musculoskeletal, and ocular symptoms (114, 130, 132, 133). In a comparative trial with MWS patients, canakinumab led to a 93% of remission rate compared to 75% of anakinra (134).

Canakinumab has been demonstrated to stabilize sensorineural hearing loss also in a subset of patients with DFN34 (135). Canakinumab represents nowadays the treatment of choice for CAPS patients for its efficacy, safety and flexibility, as the schedule of regimen could be modified according to the clinical response; a “window of opportunity”, corresponding to the earlier introduction of the drug, could be necessary to prevent long-term complications and sequelae, especially in the most severe phenotypes.

### Ongoing clinical trial in CAPS: Tranilast and DFV890

Tranilast is a tryptophan analogue which has been used for its ability to reduce mast cells activity in IgE-mediated diseases with a good tolerability; it has been recently discovered that it could inhibit NLRP3 inflammasome assembly and activation both *in vitro* and *in vivo* CAPS mouse models, in which orally administration significantly reduced mutant mice mortality rate (136). Currently a single-arm prospective study (NCT03923140) in China is recruiting CAPS patients to evaluate tranilast efficacy on clinical and inflammatory markers of the disease (137).

A single arm, phase II study (NCT04868968) is currently recruiting FCAS patients to assess the safety, tolerability, and clinical efficacy of DFV890, a novel NLRP3 inhibitor (138).

Up to date, these are the only active clinical trials exploring new treatments in CAPS patients.

### Novel small molecules targeting NLRP3 inflammasome: Evidence from CAPS mouse models

A plethora of small molecules is being investigated to inhibit NLRP3 inflammasome pathway at different phases (e.g., assembly, activation), but just a limited number of these molecules has been assessed in mouse models of CAPS. One of the first compounds evaluated in both MWS and FCAS mouse model was β-hydroxybutyrate, a ketone body physiologically produced as an ATP alternative source during fasting; it has been showed to block NLRP3 inflammasome *in vitro* and, when physiologically produced following ketogenic diet in CAPS mutant mice, it reduced neutrophilia and macrophage activation compared to the mutant mice fed with non-ketogenic diet (139).

MCC950, a selective IL-1β processing inhibitor, is a promising compound that showed its efficacy both *in vitro* than *in vivo* in CAPS mouse models: it can improve the survival rate of CINCA/NOMID mutant mice and inhibit IL-1β secretion from PMBCs of MWS patients in a dose dependent manner (140). MCC950 efficacy and safety is currently being assessed in several pre-clinical studies regarding NLRP3-mediated inflammatory diseases other than CAPS with promising results (141). Very similar results have been achieved with NLRP3 inhibitor OLT1177 (dapansutrile), which promoted a reduction of IL-1β secretion from PBMCs of two CAPS patients and blocked NLRP4 inflammasome activity in various NLRP3-related conditions (119, 142).

Interestingly, a new knock-in CAPS mouse model from Bertoni and colleagues showed that proton pump inhibitors (PPI), an old and widely available drug, administered intravenously from 2-to-5 fold the standard human dose, could increase the survival rate of mutant mice when compared to the control group, leading to remission of cutaneous rash, decrease in inflammatory markers, and even prevention of renal amyloidosis (143). The anti-inflammatory role of PPI is not fully understood, but it has been hypothesized that it could induce post-translation modifications which inhibit NLRP3 inflammasome activity, in a comparable way to the selective NLRP3 inhibitor MCC90 (143).

Even though promising, as showed in the above-mentioned results, most of these compounds have not been evaluated in humans and particularly in CAPS patients. Further clinical studies are necessary to confirm their efficacy and safety profile, paving the way for increasingly target therapies that can ensure a better quality of life for patients.

## Conclusion

The NLRP3 inflammasome plays a crucial role in the modulation of the innate immune system, protecting the host from a range of stimuli. After the formation of the NLRP3 inflammasome complex, caspase-1 promotes the maturation and the subsequent release of pro-inflammatory cytokines, mainly IL-1β and IL-18, that in turn, continue the inflammatory signaling pathway recruiting immune cells to fight infections or tissue damage. A dysfunctional activation of the NLRP3 inflammasome leads to chronic inflammation, a common pathogenetic link between several conditions, including autoinflammatory, autoimmune, and infectious states, as well as degenerative and metabolic diseases and tumorigenesis.

Gain-of-functions mutations in *NLRP3* gene are causative of cryopyrin associated periodic syndrome (CAPS), an inherited autoinflammatory disorder encompassing a continuum of three phenotypes, but new evidence have shown the causative role of NLRP3 in other autoinflammatory syndromes, such as deafness autosomal dominant 34 and keratitis fugax hereditaria, thus expanding the clinical and genetic spectrum of NLRP3-associated autoinflammatory diseases. Thus, NLRP3, given its pleiotropic role in inflammation and the considerable progress in its functional characterization, has become an increasingly interesting target in the field of drug discovery, especially for patients with CAPS. Satisfactory results have already been achieved, especially with the IL-1 antagonists but more research is required to further our understanding of the complex biology of the NLRP3 inflammasome and consequently of the selective NLRP3 inhibitors with a promising clinical efficacy and safety profile.

Therefore, whether it is possible to inhibit the downstream factors of NLRP3 inflammasome and its signaling pathways is expected to provide effective therapeutic options as well as new potential future targeted therapies for CAPS and other NRLP3-associated autoinflammatory diseases.

Moreover, due to the clinical overlap, as sometimes observed in these diseases, we suggest implementing the use of next generation sequencing approach in clinical practice to formulate an appropriate clinical and genetic diagnosis, thus leading to an early treatment and adequate follow-up.

## Author contributions

CM and MR conceived the ideas, concepts, and wrote the paper. AM revised the entire manuscript. All authors contributed to the article and approved the submitted version.

## Funding

Italian Ministry of Health, Current research – Fondazione IRCCS Ca’ Granda Ospedale Maggiore Policlinico, Milano.

## Acknowledgments

**Figures 1**, **2** were created with BioRender.com.

## Conflict of interest

The authors declare that the research was conducted in the absence of any commercial or financial relationships that could be construed as a potential conflict of interest.

## Publisher’s note

All claims expressed in this article are solely those of the authors and do not necessarily represent those of their affiliated organizations, or those of the publisher, the editors and the reviewers. Any product that may be evaluated in this article, or claim that may be made by its manufacturer, is not guaranteed or endorsed by the publisher.
